# The Effect of Cholesterol on the Long-Range Network of Interactions Established among Sea Anemone Sticholysin II Residues at the Water-Membrane Interface

**DOI:** 10.3390/md13041647

**Published:** 2015-03-25

**Authors:** Sara García-Linares, Ida Alm, Terhi Maula, José G. Gavilanes, Johan Peter Slotte, Álvaro Martínez-del-Pozo

**Affiliations:** 1 Department of Biochemistry and Molecular Biology I, Complutense University, 28040 Madrid, Spain; E-Mail: saraglinares@gmail.com; 2 Biochemistry, Department of Biosciences, Åbo Akademi University, 20520 Turku, Finland; E-Mails: iasalm@tutu.fi (I.A.); terhi.kuru@abo.fi (T.M.); jpslotte@abo.fi (J.P.S.)

**Keywords:** actinoporin, equinatoxin, sphingomyelin, pore-forming toxin, sphingomyelin

## Abstract

Actinoporins are α-pore forming proteins with therapeutic potential, produced by sea anemones. Sticholysin II (StnII) from *Stichodactyla helianthus* is one of its most extensively characterized members. These proteins remain stably folded in water, but upon interaction with lipid bilayers, they oligomerize to form a pore. This event is triggered by the presence of sphingomyelin (SM), but cholesterol (Chol) facilitates pore formation. Membrane attachment and pore formation require changes involving long-distance rearrangements of residues located at the protein-membrane interface. The influence of Chol on membrane recognition, oligomerization, and/or pore formation is now studied using StnII variants, which are characterized in terms of their ability to interact with model membranes in the presence or absence of Chol. The results obtained frame Chol not only as an important partner for SM for functional membrane recognition but also as a molecule which significantly reduces the structural requirements for the mentioned conformational rearrangements to occur. However, given that the DOPC:SM:Chol vesicles employed display phase coexistence and have domain boundaries, the observed effects could be also due to the presence of these different phases on the membrane. In addition, it is also shown that the Arg51 guanidinium group is strictly required for membrane recognition, independently of the presence of Chol.

## 1. Introduction

Sea anemones are a group of benthic marine animals which secrete various toxins [1] including a group of small and basic α-pore forming proteins known as actinoporins [2]. These actinoporins form cation-selective pores on the cell membranes, causing colloid-osmotic shock that leads to cells death [3,4,5]. They are believed to participate in anemone functions such as predation, defense, and digestion, and have been shown to be lethal to small crustaceans, mollusks, fish [6], and parasites [7]. All known actinoporins display high sequence identity and appear as multigene families [8,9]. However, only four of them have been characterized in deep detail: Equinatoxin II (EqtII) from *Actinia equina* [8], Sticholysins I and II (StnI and StnII) from *Stichodactyla helianthus* [10,11], and Fragaceatoxin C (Fra C) from *Actinia fragacea* [12]. Like many other marine toxins, actinoporins show some therapeutic potential, including different pharmacological effects, presumable anticancer activities, and use in the construction of specific immunotoxins [1,7,13,14,15,16,17,18].

In addition to their potential as therapeutic drugs, actinoporins have gained remarkable attention because they show a singular behavior at the water–lipid membrane interface. In aqueous solution they remain stably folded, but they become integral membrane structures upon interaction with lipid bilayers, oligomerizing to form pores [10,11,19]. It is widely accepted that the bilayers targeted must contain sphingomyelin (SM) and/or display phase coexistence [20,21,22,23,24,25]. In fact, the effect of not only SM but also Chol on the membrane pore-forming ability of StnII has been thoroughly studied [21,22,25,26,27,28,29]. According to those results, it is now quite clear that the presence of Chol eases the formation of pores by StnII, a conclusion which is in agreement with the coexistence of Chol and SM in biological membranes [27,30,31,32,33]. However, what still remains poorly studied is the nature of the protein determinants which explain this effect. To answer this question, we have studied a battery of different StnII mutants affecting different protein regions presumably involved in pore formation.

**Figure 1:**
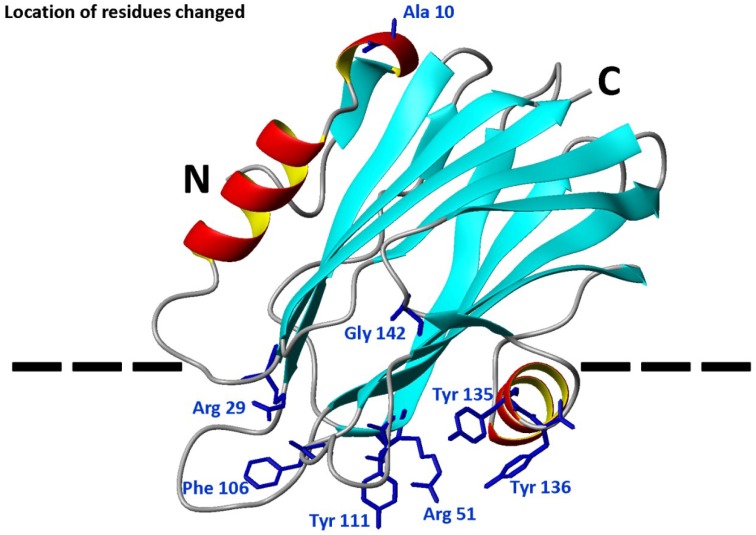
Diagram of the three-dimensional structure of StnII indicating the location of the eight mutated positions: Ala10, Arg29, Arg51, Phe106, Tyr111, Tyr135, Tyr136, and Gly142. The different elements of ordered secondary structure, as well as the *N*- and *C*-terminal ends, are also indicated. The dotted line is a representation of the membrane surface. The diagram was constructed from the atomic coordinates deposited to the PDB (Protein Data Bank, reference 1GWY for StnII). The image was generated by the MolMol Program [42].

The water-soluble structure of StnII is known in detail [34]. It folds as a β-sandwich motif composed of 10 β-strands flanked by two α-helices which interact with both sides of the β-sandwich (Figure 1). One of these helices (α1) is located near the *N*-terminal end. In fact, the first 30 residues appear to be able to adopt alternative conformations without disrupting the fold of the β-sandwich [35]. This feature, altogether with the amphipathic character of this stretch, seems to be extremely important for the final functionality of the pore, since the α1 helix has been proposed to extend and further insert into the membrane to form the pore walls [17,36,37]. The most recent model explaining the mechanism of actinoporins’ pore formation [17,37,38,39] assumes a toroidal protein-lipid structure without a well-defined fixed stoichiometry [17,38,39,40], although an alternative model has been proposed for Fra C [41]. Nevertheless, it is reasonably well-proven that insertion of the *N*-terminus into the membrane takes place in anon-coordinate way, shortly after the binding of the toxin and before their oligomerization into the final pore [37]. In addition to this *N*-terminal α-helix, three more regions of the structure seem to be especially important from a functional point of view: a phosphocholine (POC) binding site, a cluster of aromatic residues, and an array of basic amino acids [11].

The work presented here studies the effect of Chol on the membrane-interacting behavior of eight different StnII mutants affecting the stretch of the 30 first residues (A10P and R29Q), the aromatic cluster (F106L, Y111N, Y135F, and Y136F), the POC binding site (R51Q, Y111N, Y135F, and Y136F), and a residue involved in maintaining the pore-competent state of protein oligomerization (G142A).

## 2. Results

### 2.1. Protein Purification and Characterization

All proteins used in the study were purified to homogeneity according to their behavior in SDS-PAGE. Their amino acid compositions were consistent with the introduced mutations. The calculated E^0.1%^ (280 nm, 1 cm) values were also in good agreement with the amino acid changes made (Table 1). All far-UV CD spectra of the individual mutants were indistinguishable from that corresponding to the wild-type StnII (data not shown). In summary, all eight mutants retained the overall native water-soluble conformation.

The hemolytic activity was diminished in all the mutants studied (Table 1). This may not be a surprise, given that all the residues changed are presumably involved in essential steps for the formation of the final functional pore [10,11,37]. This effect was especially evident for mutants R29Q, Y111N, and G142A (Table 1) [29,43,44].

Stability analyses also revealed the importance of some of these residues (Arg29 and Gly142) in maintaining the protein conformation as deduced from the large decrease of the *T*_m_ values for R29Q and G142A mutant variants (Table 1). It is also remarkable that substitution of Tyr111 by Asn and Tyr136 by Phe produced mutant proteins with higher *T*_m_ values (Table 1). All mutants studied showed values high above the temperatures used along the study and, therefore, the results described below should not be attributed to thermal denaturing effects.

**Table 1:** Structural and functional parameters of the proteins used in the study.

StnII Variant	E^0.1%^ (280 nm, 1 cm)	*T*_m_ (°C)	Relative Hemolytic Activity ^c^
Wild-type	2.54 ^a^	67 ^a^	1.00
A10P	2.69 ^a^	66 ^a^	0.26 ^a^
R29Q	2.54 ^a^	60 ^a^	<0.10 ^a^
R51Q	2.38	67	0.30
F106L	2.62 ^a^	66 ^a^	0.39 ^a^
Y111N	2.58 ^a^	70 ^a^	<0.10 ^a^
Y135F	2.47	66	0.26
Y136F	2.66	69	0.26
G142A	2.30 ^b^	61 ^b^	0.13 ^b^

^a^ [29]; ^b^ [44]; ^c^ Relative hemolytic activity calculated as HC_50_(WT)/HC_50_(mut).

### 2.2. Protein Binding to the Two Different Lipid Model Vesicles Employed

**Figure 2:**
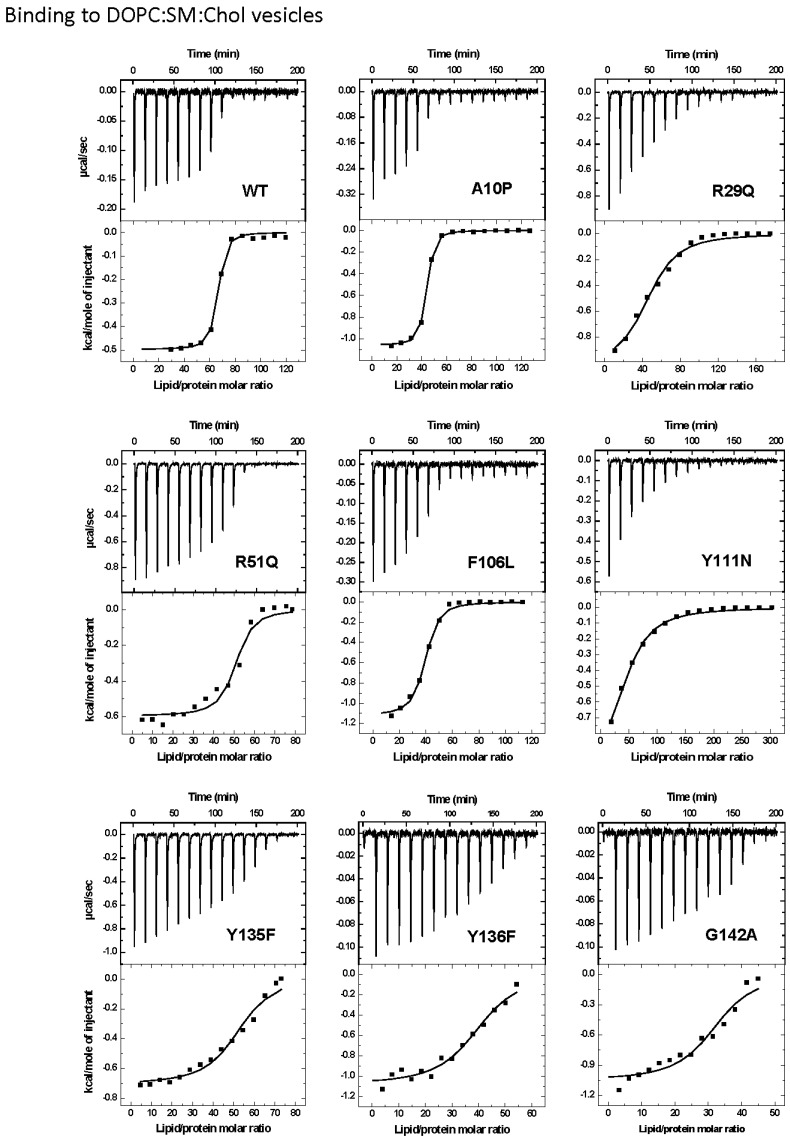
Binding of StnII and its mutants to DOPC:SM:Chol (1:1:1) 100-nm-diameter vesicles studied by ITC. Reactant concentrations were as follows: WT (P_0_ = 1.2 μM, L_0_ = 0.56 mM), A10P (P_0_ = 1.1 μM, L_0_ = 0.55 mM), R29Q (P_0_ = 2.3 μM, L_0_ = 1.8 mM), R51Q (P_0_ = 10.4 μM, L_0_ = 3.6 mM), F106L (P_0_ = 1.1 μM, L_0_ = 0.53 mM), Y111N (P_0_ = 1.2 μM, L_0_ = 1.6 mM), Y135F (P_0_ = 10.4 μM, L_0_ = 3.4 mM), Y136F (P_0_ = 1.0 μM, L_0_ = 0.25 mM), and G142A (P_0_ = 1.2 μM, L_0_ = 0.25 mM). Binding isotherms were adjusted to a model in which protein membrane binding involves the participation of “*n*” lipid molecules. The c values (c = K × P_0_) for all the graphs are in the range 1–1000.

**Figure 3:**
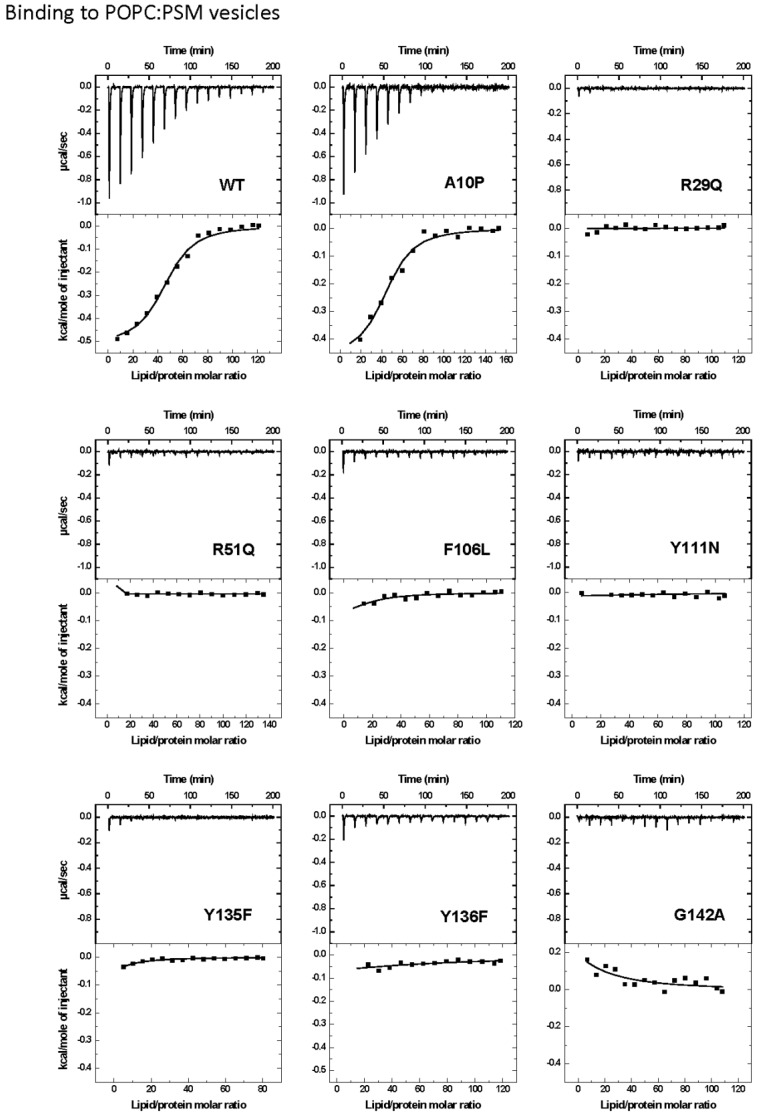
Binding of StnII and its mutants to POPC:PSM (4:1) 100-nm-diameter vesicles studied by ITC. Reactant concentrations were as follows: WT (P_0_ = 10.0 μM, L_0_ =5.4 mM), A10P (P_0_ = 8.0 μM, L_0_ = 5.4 mM), R29Q (P_0_ = 9.9 μM, L_0_ = 4.8 mM), R51Q (P_0_ = 10.5 μM, L_0_ = 6.3 mM), F106L (P_0_ = 9.1 μM, L_0_ = 4.9 mM), Y111N (P_0_ = 10.1 μM, L_0_ = 5.1 mM), Y135F (P_0_ = 10.3 μM, L_0_ = 3.7 mM), Y136F (P_0_ = 9.6 μM, L_0_ = 5.1 mM), and G142A (P_0_ = 9.4 μM, L_0_ = 5.0 mM). Binding isotherms were adjusted to a model in which protein membrane binding involves the participation of “*n*” lipid molecules. Given the low affinity of most of the mutants for the vesicles assayed, in this case only the c values (c = K × P_0_) for WT and A10P are in the range 1–1000.

ITC was used to measure the interaction of the StnII mutants with DOPC:SM:Chol (1:1:1) or POPC:PSM (4:1) vesicles (Figure 2 and Figure 3). The interaction of wild-type StnII with POPC:PSM (4:1) vesicles in the presence of low amounts of Chol has been recently described [27]. In the present work DOPC:SM:Chol (1:1:1) vesicles were chosen because they are considered a model of coexistence between liquid-disordered and liquid-ordered phases [31,45,46,47]. All mutants studied were observed to bind to the DOPC:SM:Chol (1:1:1) vesicles (Figure 2) although with evident differences in terms of relative membrane binding affinity values (Table 2). Thus, while A10P still bound with almost identical affinity to the wild-type StnII, and F106L showed only a reduction of about 2.5-fold (Table 2), all the other mutants displayed values about one to two orders of magnitude smaller. The most dramatic differences in affinity were observed for the R29Q, R51Q, Y111N, and Y135F mutants (Table 2). Binding to the POPC:PSM (4:1) vesicles was about 100-fold lower than to the Chol-containing ones for wild-type StnII (Table 2) and most of the mutants did not produce a detectable signal in these experiments (Figure 3). Only binding of the A10P protein variant was measurable, showing again a very similar relative binding affinity to the wild-type protein (Table 2).

**Table 2:** Binding of StnII protein variants to DOPC:SM:Chol (1:1:1) and POPC:PSM (4:1) vesicles studied by ITC. The interaction of some mutants with POPC:PSM (4:1) vesicles was too weak to be detected by ITC in the conditions used for these experiments (see Figure 3) (data not shown in the Table).

DOPC:SM:Chol (1:1:1)						
StnII Variant	*n*	K × 10^−8^ (M^−1^)	ΔG (kcal/mol)	ΔH (kcal/mol)	ΔS (cal·mol^−1^·K^−1^)	Relative Membranebinding ^a^
WT	39 ± 4 ^b^	1.700 ± 0.900 ^b^	−9.1 ± 0.5 ^b^	−44.0 ± 3.0 ^b^	−115.0 ± 9.0 ^b^	1.000 ^b^
A10P	37 ± 4 ^b^	1.900 ± 0.900 ^b^	−9.3 ± 0.4 ^b^	−39.0 ± 6.0 ^b^	−99.0 ± 20.0 ^b^	1.180 ^b^
R29Q	51 ± 8 ^b^	0.031 ± 0.002 ^b^	−6.7 ± 0.2 ^b^	−45.0 ± 3.0 ^b^	−129.0 ± 8.0 ^b^	0.014 ^b^
R51Q	49 ± 2	0.140 ± 0.070	−7.6 ± 0.2	−29.0 ± 1.0	−72.0 ± 4.0	0.082
F106L	36 ± 3 ^b^	0.600 ± 0.100 ^b^	−8.6 ± 0.1 ^b^	−37.0 ± 7.0 ^b^	−94.0 ± 24.0 ^b^	0.380 ^b^
Y111N	46 ± 7 ^b^	0.025 ± 0.004 ^b^	−6.6 ± 0.1 ^b^	−47.0 ± 2.0 ^b^	−134.0 ± 4.0 ^b^	0.012 ^b^
Y135F	51 ± 2	0.039 ± 0.013	−6.8 ± 0.2	−36.0 ± 2.0	−99.0 ± 6.0	0.023
Y136F	40 ± 1	0.270 ± 0.080	−8.1 ± 0.1	−43.0 ± 1.0	−117.0 ± 4.0	0.160
G142A	32 ± 1	0.250 ± 0.100	−8.2 ± 0.1	−33.0 ± 1.0	−83.0 ± 4.0	0.150
**POPC:PSM (4:1)**						
WT	45 ± 2	1.5 ± 0.3	−6.3 ± 0.1	−23.0 ± 1.0	−57.0 ± 3.0	1.000
A10P	44 ± 3	1.2 ± 0.4	−6.2 ± 0.2	−20.0 ± 2.0	−48.0 ± 6.0	0.800

^a^ [n(WT) × K(mut)]/[n(mut) × K(WT)] as explained in [29]; ^b^ [29].

### 2.3. Pore Formation

**Figure 4:**
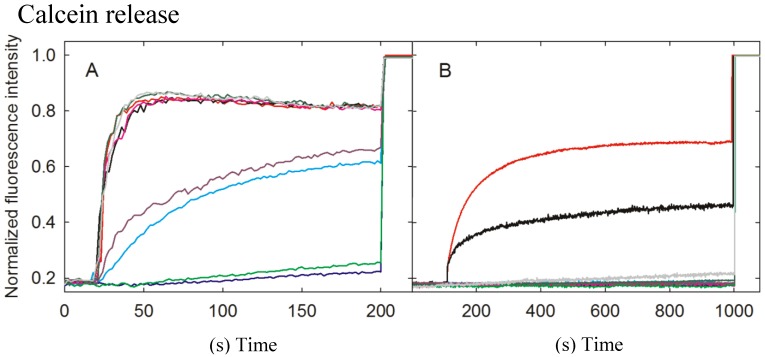
Release of calcein entrapped in DOPC:SM:Chol (1:1:1) (**A**) or POPC:PSM (4:1) (**B**) vesicles by wild-type StnII (red) or its A10P (black), R29Q (light blue), R51Q (dark blue), F106L (magenta), Y111N (light green), Y135F (purple), Y136F (dark green), and G142A (gray) mutants. Calcein-entrapped vesicles were prepared with extrusion and release was measured at 23 °C. All intensities were normalized. Towards the end of the measurement (at 200 s in A and 800 s in B), Triton X-100 was added to dissolve the LUVs and release all calcein. The results shown are representative of two different independent experiments.

Pore formation was monitored by recording the fluorescence emission of calcein released from lipid model vesicles. These vesicles are only a simplified version of a real membrane. This would explain why, in general, actinoporins seem to be more effective against erythrocytes. However, this approach is extremely useful because it allows the determination of different biophysical parameters and, most of all, the dissection of the individual roles of different types of lipid molecules, as is the case in this work. Within this idea, wild-type StnII and the A10P, F106L, Y136F, and G142A variants displayed an indistinguishable kinetic of calcein release from DOPC:SM:Chol (1:1:1) vesicles (Figure 4A). The R29Q and Y135F mutants were able to induce calcein leakage but exhibited a largely decreased velocity constant. The R51Q and Y111N mutants, on the other hand, were unable to release the entrapped fluorophore (Figure 4).

For POPC:PSM (4:1) vesicles, only the A10P mutant variant was able to induce calcein release, although to a lower extent than the wild-type protein (Figure 4B). This would be explained by the conformational stiffness introduced in this StnII mutant by the substitution of an Ala residue by Pro that would hamper pore formation without loss of binding affinity [29].

## 3. Discussion

**Figure 5:**
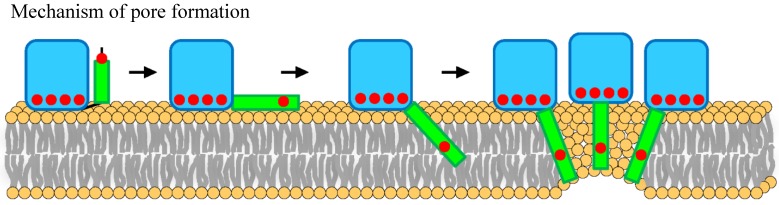
Illustration representing the pore-forming mechanism of actinoporins. Once the protein is attached to the membrane, the *N*-terminal α1 helix extends and inserts into the bilayer. Finally, oligomerization conduces to a final toroidal pore. The blue shape represents the β-sandwich core. The green dots represent the 30 first residues before and after the extension of the α1 helix extension. Red dots represent the approximate location of the residues mutated in the work presented here. This figure has been adapted from [37].

Pore formation by actinoporins is the result of a series of well coordinated events. The recently refined model of this mechanism [37,39,40] predicts that, after an extremely fast toxin binding to the membrane, major conformational changes occur, resulting in the simultaneous presence of several distinct membrane-bound protein forms (Figure 5). These conformational changes involve long-distance rearrangements [48,49] which complicate the interpretation of the effects produced by individual mutations, especially if these changes affect the protein face involved in recognizing the membrane. According to the mentioned mechanism, one of the first distinct conformational species to appear would be represented by a molecule in which an extended *N*-terminal α1 helix would lie more or less parallel to the membrane surface [50]. Then, this elongated α1 helix would be inserted into the membrane, a step which would be faster than the oligomerization step [37]. Finally, functional pore formation would take place as the result of a mechanism which begins with the formation of dimers and ends in a toroidal protein-lipid structure (Figure 5) lacking a well-defined fixed stoichiometry [17,38,39,40]. A stable prepore structure is not required according to this model, in clear contrast to β-pore forming toxin behavior [51,52]. However, an alternative non-toroidal nonameric pore based on a detergent-containing crystalline structure, has been also proposed for the actinoporin Fra C [41]. Independently of the pore formation mechanism, it is well-established that Chol enhances pore formation by actinoporins not only because induces the formation of raft-like domains but also because, even at low concentrations, it still affects the physical state of SM [21,22,26,27,28,29]. The results presented here reveal the influence of this lipid on the essential long-distance rearrangements required for pore-formation, since the StnII variants that were studied harbor mutations that affect residues in protein regions with assigned key roles in pore formation (Figure 1 and Figure 5).

### 3.1. StnII Mutants Affecting the 30 First Residues Sequence Stretch

StnII Ala10 is located at the edge of the region thought to become α-helical to form the pore [50]. Thus, StnII A10P contains a mutation which is located far away from the membrane binding surface (Figure 1 and Figure 5) and therefore does not participate directly in the long-distance rearrangements needed for membrane recognition. This mutation has been shown to provide enough conformational stiffness so as to hamper the required α1 helix extension [29,43,53] (Figure 5). This is in good agreement with the behavior of a double cysteine mutant of EqtII, which was also designed to increase the stiffness of this protein region [54]. It was then proposed that neither the presence of the *N*-terminal stretch region nor the conformational changes occurring along this sequence after membrane binding would provide higher affinity of StnII for the bilayer. Binding of A10P was not substantially affected by the presence or absence of Chol. However, hemolysis and calcein leakage activity in the absence of Chol were clearly diminished (Table 1 and Table 2; Figure 4B), thus suggesting that pore formation, but not membrane recognition, is impaired in this mutant. On the other hand, like the wild-type protein, A10P was observed to bind with greater affinity to Chol-containing lipid vesicles (Table 1 and Table 2), which explains the differences observed between both types of vesicles in terms of calcein leakage. Thus, A10P could be considered a reference for efficient protein binding without pore formation. The presence of Chol (Figure 4A) allows leakage of water soluble solutes even in the absence of a completely extended *N*-terminal α-helix, a result that would reinforce the hypothesis in favor of the toroidal nature of the pore [17,38,39].

The other residue mutated along this polypeptide region, Arg29, is a conserved amino acid [11] located at the *C*-terminal end of this 30 residues long protein stretch (Figure 1). This residue has been shown to be crucial because of its involvement in membrane recognition and also in sustaining the necessary conformational changes leading to pore formation [29]. A rotation between StnII Ser28 and Arg29 might occur after membrane binding [34], inducing these conformational changes. In addition, Arg29 and Phe106 establish a cation-π interaction, which is needed to provide the protein with the right conformational flexibility [43]. This interaction is not possible in the R29Q mutant, which results in an increased conformational freedom of this region that, in turn, greatly distorts the distribution of the electrostatic potential along the surface of the protein face involved in recognizing the membrane [48]. Relative membrane binding affinity for the DOPC:SM:Chol (1:1:1) was greatly diminished (Table 2; Figure 2), in agreement with less hemolytic (Table 1) and calcein release activities, (Figure 4). In the absence of Chol, binding and calcein leakage were not detectable (Figure 3 and Figure 4B). Altogether, these results reveal a key role for Chol in the correct binding and positioning of the region around StnII Arg29 on the membrane in order to produce a functional pore.

### 3.2. StnII Mutants Affecting the Aromatic Cluster

The mutations affecting the aromatic cluster of StnII were F106L, Y111N, Y135F, and Y136F. NMR analysis of the aromatic resonances of the equivalent StnI groups in the presence of dodecylphosphocholine micelles revealed a high motional flexibility of StnI Tyr-136 and Tyr-137 (Tyr 135 and 136 in StnII) [55]. From this point of view, given that the hydrophobicity of the side chain of these two residues is maintained in the mutants, it can be explained why they still interact fairly well with the Chol containing vesicles (Figure 2 and Figure 4A) while they are completely unable to maintain the specific contacts in the absence of Chol (Figure 3 and Figure 4B; see also below). Interestingly, StnITyr-112 (Tyr111 in StnII) was not affected by the micellar media [55]. Detailed inspection of the water soluble forms of EqtII and StnII reveals that the loop containing this residue (segment spanning StnII Tyr108 to Tyr111) is completely disordered and displays high conformational flexibility [34,35,56]. In the crystal structure of the StnII:POC complex [34], the aromatic ring of Tyr111 is pointing towards the POC moiety after a probable conformational change from its exposed POC-free state [55]. Indeed, it has also been proposed that Tyr111 would induce a necessary disorder in exposed hydrophobic chains to promote their interaction with the membrane [48]. Finally, as stated above, Phe106 and Arg29 establish an interaction which may function like a switch to turn on the conformational changes needed for protein binding to the membrane [43,48].

Taking all this into account, the results presented here agree with the existence of the aforementioned network of long-range interactions, given that any of the single mutations studied rendered protein variants completely unable to interact with the POPC:PSM (4:1) vesicles (Figure 3 and Figure 4). This situation changed significantly in the presence of Chol (Figure 2 and Figure 4) since some of the residues involved are also part of the POC-binding site, as explained below.

### 3.3. StnII Mutants Affecting the POC-Binding Site

The StnII mutants affecting the POC-binding site studied herein were R51Q, Y111N, Y135F, and Y136F. Three of these mutated amino acids are involved in the aromatic cluster, and all four participate in specific interactions with the POC moiety and/or the 2NH and 3OH groups of SM [24,29,34,41,43,48,55,57]. Therefore, these four mutants displayed diminished hemolytic activities (Table 1) and lacked the ability to interact with PSM containing vesicles in the absence of Chol (Table 2; Figure 3 and Figure 4). However, they behave differently when studied against DOPC:SM:Chol (1:1:1) vesicles (Table 2; Figure 3 and Figure 4) and could be ascribed to three different behavior patterns.

First, in the presence of Chol, Y136F was quite similar to the wild-type StnII in terms of membrane affinity (Figure 2) and calcein release activity (Figure 4A) but was not able to interact with the POPC:PSM (4:1) vesicles (Figure 3). These results agree with the role assigned to Tyr136 in interacting specifically with the phosphate oxygens of the POC moiety [24], contributing to its stabilization within the POC-binding pocket. In the absence of Chol, this interaction would then be essential for maintaining the SM molecule at the right position, and therefore this mutant failed in binding efficiently enough to the vesicles.

The second pattern would correspond to the Y135F mutant. Tyr135 is a residue which has been related to interactions with the SM 2NH and 3OH groups through the phenolic hydroxyl group [24]. Both interactions would be absent in the mutant assayed. This would explain the obtained results in terms of lower hemolytic activity (Table 1) and much lower relative membrane binding affinity (Table 2) as well as the dampened calcein release properties (Figure 4A) against DOPC:SM:Chol (1:1:1) vesicles. However, this mutant was completely unable to bind to the POPC:PSM (4:1) vesicles, and therefore it can be inferred that the interactions with the SM molecule involving Tyr135 are enhanced in the presence of Chol.

Regarding the third pattern, the last two mutants studied, R51Q and Y111N, displayed quite low hemolytic activity (especially Y111N) and a rather low affinity for both types of vesicles studied (Table 2).They were also completely inactive in the calcein release experiments (Figure 4). It has been proposed that the POC phosphate moiety could be further stabilized by the cationic side chain of Arg51 [24]. Thus, these results would also support the abovementioned role of Tyr111 in the observed conformational change from its exposed free state to the POC-bound complex [34]. The Y111N mutant would not be able to provide the required interactions and therefore would fail to produce a functional pore even in the presence of Chol (Figure 4A). Finally, according to the present results, it can also be stated that the interaction provided by the side chain of StnII Arg51 is strictly required for membrane recognition independently of Chol.

### 3.4. StnII Mutant Affecting the Pore-Competent State of Protein Oligomerization

**Figure 6:**
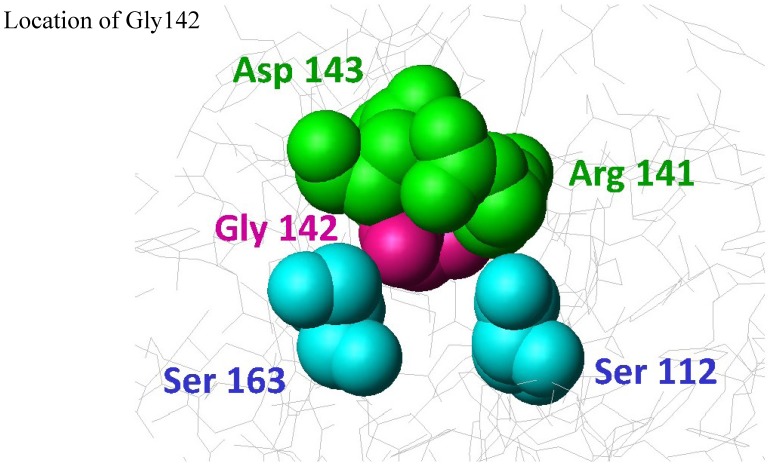
Close-up diagram representation of the residues presumably affected by substitution of StnII Gly142 (red) by an Ala residue: Ser112 (blue), Arg141 (green), Asp143 (green), and Ser163 (blue). Image was generated by the MolMol Program [42].

StnII Gly142 belongs to a conserved RGD motif which has been related to maintaining the protein in the correct aggregation competent state, not only in its water-soluble state, but also in its membrane bound configuration [44]. Therefore, it would not be a residue directly involved in membrane recognition, nor in pore formation (Figure 1). In good agreement with this hypothesis, in the presence of Chol, both the G142A mutant and the wild-type protein showed similar behaviors in terms of membrane binding (Table 2; Figure 2) and calcein release activity (Figure 4A). However, this mutant failed to bind to the POPC:PSM (4:1) vesicles (Figure 3). Inspection of the relative orientation of Gly142 within the StnII three-dimensional structure (Figure 1 and Figure 6) suggests how this residue could also be involved in the required long-distance rearrangements [48,49] for its correct attachment to the membrane. The presence of an additional obstructing methyl group in the G142A mutant would distort not only a well-established ionic interaction between Arg141 and Asp143, but also the stabilization of Gly142 by the hydroxyl group of Ser163 (Figure 6). Mutation to Ala would then interfere with these rearrangements, leading to a more aggregation-prone protein that is nonetheless unable to bind to the membrane in a pore-competent state. This effect would be especially evident in the absence of Chol.

## 4. Experimental Section

### 4.1. Materials

1-Palmitoyl-2-oleoyl-*sn*-glycero-3-phosphocholine (POPC), 1,2-dioleoyl-sn-glycero-3-phosphocholine (DOPC), cholesterol (Chol), and porcine brain sphingomyelin (SM) were obtained from Avanti Polar Lipids. Palmitoyl-SM (PSM) was purified from egg SM using preparative HPLC on a reverse phase (C18) column, as described previously [58,59]. Calcein was from Fluka/Sigma-Aldrich (St. Louis, MO 63103, USA). Sephacryl S200HR was obtained from GE Healthcare. The cDNA coding for the StnII mutants were obtained by the overlap extension mutagenesis method [60], also as described [61]. Wild-type StnII and all the mutants used in this study were produced in an *E. coli* expression system, and purified as described previously [29,43,61].The homogeneity of all protein samples used was analyzed by SDS/PAGE and amino acid analysis after acid hydrolysis of the proteins (5.7 M HCl, 24 h, 110 °C). These amino acid analyses were performed on a Biochrom 20 automatic analyzer (Pharmacia, Cambridge, UK).

### 4.2. Spectroscopic Characterization

Spectroscopic characterization was essentially performed as previously described [29,44,49,62,63,64]. Absorbance measurements were carried out on a Uvikon 930 spectrophotometer (Kontron Instruments, Madrid, Spain). The individual E^0.1%^ (280 nm, 1 cm) coefficients were calculated for every single protein species as described [21,29,44], using their amino acid compositions and specific UV-absorption spectra [9,21,65]. Far-UV circular dichroism (CD) spectra were obtained on a Jasco 715 spectropolarimeter at50 nm/min scanning speed. Optical path cells of 0.1 cm were employed. Proteins were dissolved in 15 mM MOPS buffer, pH 7.5, containing 100 mM NaCl (0.2 mg/mL protein concentration). At least four spectra were averaged to obtain the final spectrum. CD measurements were also employed to study the thermal stability of the mutants, as described before [29,49,62]. *T*_m_ values correspond to the temperature at the midpoint of the monophasic thermal denaturation transition.

### 4.3. Hemolysis Assay

Hemolysis assays were performed in 96-multiwell plates as previously described [29,61]. Briefly, erythrocytes from heparinized sheep blood were washed in 10 mM Tris buffer, pH 7.4, containing 0.145 M NaCl, to a final OD_655_ of 0.5 when mixing equal volumes of the cell suspension and buffer. The hemolysis was followed as a decrease in OD_655_ after addition of the erythrocyte suspension to twofold serial dilutions of the proteins. An Expert 96 microplate reader (AsysHitech, GmbH, Eugendorf, Austria) was employed to measure OD_655_. The value obtained with 0.1% (w/v) Na_2_CO_3_ was considered 100% hemolysis. HC_50_ is the protein concentration required to produce 50% hemolysis.

### 4.4. Binding of StnII to Bilayer Membranes

Binding was measured using isothermal titration calorimetry (ITC) as described before [24,29,44], using a VP-ITC calorimeter (MicroCal). Briefly, protein solutions at 10 μM were titrated by injection of 20 μL aliquots of lipid suspensions (phospholipid concentration: 5 mM). Binding isotherms were adjusted to a model were the protein binds the membrane involving “n” lipid molecules [29].

### 4.5. Calcein Leakage Assay

Calcein-entrapped POPC:PSM (4:1) or DOPC:SM:Chol (1:1:1) large unilamellar vesicles (LUVs) were prepared as described [27] by extrusion through 200 nm filters (Nucleopore, Whatman) at 37 °C, or 60 °C if Chol was not present. Briefly, the desired lipids were mixed and dried under a stream of nitrogen. The lipids were re-dissolved in chloroform and dried again before removal of any traces of remaining solvent in vacuum for 60 min. Prior to extrusion, the dry lipid films were hydrated for 30 min at 60 °C (or 37 °C if Chol was present) in Tris buffer (10 mM Tris, 140 mM NaCl, pH 7.4) containing calcein at a concentration of 100 mM. The total lipid concentration was 1.25 mM. LUVs were separated from non-entrapped calcein by gel filtration on Sephacryl S200HR. These LUVs were used for permeabilization studies within 24 h. Phospholipid concentration was determined from P_i_ measurement [66] after elution of vesicles during isolation. The concentration of LUV phospholipids and StnII during calcein leakage experiments were about 7.5 μM and 20 nM, respectively. Emission at 550 nm was followed at 23 °C as a function of time (excitation at 480 nm). Fluorescence emission was measured with a PTI Quanta-Master spectrofluorimeter (Photon Technology International, Inc., Edison, NJ, USA). To ensure that no major spontaneous leakage occurred, the emission was measured for each sample during 5 min before addition of toxin. A steady signal level, indicating intact vesicles, was observed for all samples.

## 5. Conclusions

One major conclusion of the results presented here is that Chol is a key molecule in the correct and efficient interaction of StnII with its target membranes in order to produce a functional pore. It is now proven that this lipid molecule is even able to circumvent the impairment produced by the substitution of residues specifically involved in SM recognition and/or membrane surface attachment. Thus, although SM would definitively be the crucial molecule for specific membrane recognition, Chol would also act as a highly important partner in this interaction. At the temperatures employed in these experiments, POPC:SM (4:1) bilayers exist in a liquid disordered phase. However, the DOPC:SM:Chol vesicles employed display liquid disordered-liquid ordered phase coexistence and have domain boundaries. Therefore, the observed effects of Chol could be due not only to the reported specific influence on the SM physical state [27] but also to coexistence of different phases on the membrane. Similar effects have been reported already, if not for attachment, at least for the final formation of the pore [21,25,29,67].

It has been previously acknowledged that a series of conformational changes involving long-distance rearrangements of the residues on the protein side facing the membrane must occur for the correct attachment of StnII to the membrane surface. Substitution of individual residues involved in these rearrangements has a dramatic impact on the interaction of StnII with PSM containing vesicles in the absence of Chol, but the impact of these mutations is nearly negligible if Chol is present. Therefore, it can also be concluded that the presence of Chol would ease the requirements needed for these conformational rearrangements to occur.
